# Screening for Fabry Disease in Young Strokes in the Australian Stroke Clinical Registry (AuSCR)

**DOI:** 10.3389/fneur.2020.596420

**Published:** 2020-11-24

**Authors:** Alejandra Malavera, Dominique A. Cadilhac, Vincent Thijs, Joyce Y. Lim, Brenda Grabsch, Sibilah Breen, Stephen Jan, Craig S. Anderson

**Affiliations:** ^1^Faculty of Medicine, The George Institute for Global Health, University of New South Wales, Sydney, NSW, Australia; ^2^Stroke and Ageing Research, Department of Medicine, School of Clinical Sciences at Monash Health, Monash University, Clayton, VIC, Australia; ^3^Florey Institute of Neuroscience and Mental Health, University of Melbourne, Heidelberg, VIC, Australia; ^4^Department of Neurology, Austin Health Heidelberg, Heidelberg, VIC, Australia; ^5^Sydney Medical School, University of Sydney, Sydney, NSW, Australia; ^6^Neurology Department, Royal Prince Alfred Hospital, Sydney Health Partners, Sydney, NSW, Australia

**Keywords:** fabry disease, α-galactosidase A, screening, blood spot test, GLA gene, young stroke

## Abstract

**Introduction:** Fabry disease (FD) is an X-linked lysosomal storage disorder characterized by a deficiency or absence of alpha-galactosidase A (α-GAL A) enzyme, where stroke can be a serious complication. The aim of this study is to determine the feasibility of centralized screening for FD, among young stroke adults registered in the national Australian Stroke Clinical Registry (AuSCR).

**Methods:** The study was conducted in young (age 18 – 55 years) survivors of acute stroke of unknown etiology registered in AuSCR at hospitals in Queensland, Tasmania, New South Wales, and Victoria during 2014 – 2015; and who, at the 3-month outcome assessment, agreed to be re-contacted for future research. Descriptive analyses of case identification from responses and specific enzyme and DNA sequencing analyses were conducted for α-galactosidase A (α-GLA) from dried blood spot (DBS) testing.

**Results:** Of 326 AuSCR-identified patients invited to participate, 58 (18%) provided consent but six were subsequently unable to provide a blood sample and two later withdrew consent to use their data. Among the remaining 50 participants (median age 53 years [48 – 56 years]; 47% female), 67% had experienced an acute ischemic stroke. All males (*n* = 27) had an initial screen for α-GLA enzyme activity of whom seven with low enzyme levels had normal secondary α-GLA gene analysis. All females (*n* = 23) had genetic analysis, with one shown to have a pathogenic c.352C>T p.(Arg118Cys) missense mutation of the α-GLA gene for FD.

**Conclusions:** These findings provide logistical data for embedding a process of automated central stroke registry screening for an additional case-finding tool in FD.

## Introduction

Stroke in young adults (age <50 years) has major personal, social and economic impact (1, 2). Among the various etiologies, genetic causes are important to diagnose for counseling and consideration of available therapies (3, 4). One such condition, Fabry disease, is an X-linked lysosomal storage disorder caused by mutations in the α-galactosidase A (α-GLA A) gene, which accounts for 1 – 4% of cryptogenic strokes in younger patients (5). Absent or reduced activity of α-GAL A enzyme impairs catabolism of neutral glycosphingolipids, which progressively accumulate within the lysosomes of various cell types, including the vascular endothelium (6). Affected individuals typically show manifestations of the condition in childhood, including acroparesthesias, angiokeratoma, cornea verticillate, and hypohidrosis (7), but acute stroke can occur without any pre-existing cardiac or renal complications (8). As the condition can be easily overlooked as part of investigative work-up, when only ~1 – 2% of young strokes of unknown cause have Fabry disease (9), clinical quality assurance registries may provide an alternative mechanism to support screening efforts.

Screening for Fabry disease entails measuring α-GAL A enzyme activity in peripheral blood leukocytes or plasma but, while affected males usually have low or undetectable enzyme activity, females often have normal or only mildly reduced α-GAL A enzyme activity due to random X-chromosome inactivation (10). Thus, GLA gene sequencing is critical for diagnosing Fabry disease (11). Recently, a novel screening method has been developed for measuring α-GAL A activity from dried blood spots (DBS) on filter paper (12), but data are limited on the utility of this method for screening for Fabry disease in young strokes where the cause is unknown. Herein, we report results of a study designed to test the potential of embedding a process of screening for Fabry disease in young survivors of stroke registered in the ongoing national Australian Stroke Clinical Registry (AuSCR).

## Materials and Methods

### Study Design and Linked Dataset

This is an investigator-initiated, prospective, study using data from AuSCR for case-finding and merging of additional project generated data using patient-level data linkage. AuSCR is an ongoing, voluntary, quality assurance, clinical registry used to capture minimal data on demographics, case mix, clinical care and 3-month outcomes for consecutive patients admitted with stroke or transient ischemic attack at Australian hospitals (13). An opt-out consent process is used whereby patients, upon admission to hospital, receive an information sheet that outlines the purpose of AuSCR, type of information collected, and requirement to assess functional status and health-related quality of life by questionnaire (or telephone interview) at 3-months post-stroke. At the time of follow-up, participants are also asked if they would be willing to be re-contacted to participate in future research. Approximately 65 – 70% of the cohort complete surveys, and all registered patients have their survival status determined annually through linkage to the National Death Index by the Australian Institute of Health and Welfare.

This study pertains to individuals with the following criteria: age 18 – 55 years at time of stroke with unknown etiology; registered in AuSCR during 2014 – 2015; living at home in Queensland, Tasmania, New South Wales and Victoria; agreed to be contacted; and last known to be alive. They were sent an invitation letter (a second letter was sent to non-responders after 4 weeks) by the AuSCR Office, based in Victoria. Those who responded returned information to The George Institute for Global Health (University of New South Wales), where researchers responsible for coordinating the screening program were based. Following provision of written consent for enzymatic and genetic testing for Fabry disease, subcontracted nurses were organized to undertake home visits to obtain DBS samples and self-reported data on socio-demographic status, major comorbid conditions, vascular risk factors, and any clinical manifestations of Fabry disease.

### Screening for Fabry Disease

Eligible participants provided finger-tip blood or venous blood from an antecubital vein onto a DBS card (12) at home, facilitated by a trained nurse from an independent company that provides home-based nursing services across Australia. The DBS card was sent for enzyme and GLA gene analysis at Centogene AG laboratory, Germany, with α-GAL A enzyme activity measured in males followed by GLA gene analysis and Lyso-Gb3 levels in those with low enzyme activity. Conversely, an initial direct genetic analysis of GLA was performed in females, with Lyso-Gb3 levels only measured in those with abnormal result. Fluorometry was used to measure α-GAL A enzyme activity (normal ≥15.3 μmol/L/h) and next-generation sequencing (NGS-Illumina) was used for genetic analysis. Lyso-Gb3 levels were analyzed by liquid chromatography mass spectrometry (normal ≤1.8 ng/mL), with multiplex ligation-dependent probe amplification (MLPA). This variation of the multiplex polymerase chain reaction permits amplification of multiple targets with only a single primer pair, ideally in situations of large deletions or duplications. Results were communicated to the participants via their primary care physician (General Practitioner) or specialist, with recommendations for referral for counseling as appropriate.

### Statistical Analysis

Descriptive statistics was performed for available demographic and clinical parameters. Continuous data are shown as medians, while categorical data are given as absolute counts and proportions. Analyses were conducted using STATA version 15.1 (Stata Corporation, College Station, TX).

### Ethics Approval

Ethics approval for conduct of the study was granted by the Human Ethics Review Committee of Royal Prince Alfred Hospital (Protocol No X16-0120; HREC/16/RPAH/146). The independent AuSCR Research Task Group approved access to the AuSCR dataset (by AuSCR staff) to identify potential participants according to the study protocol.

## Results

**Figure 1:**
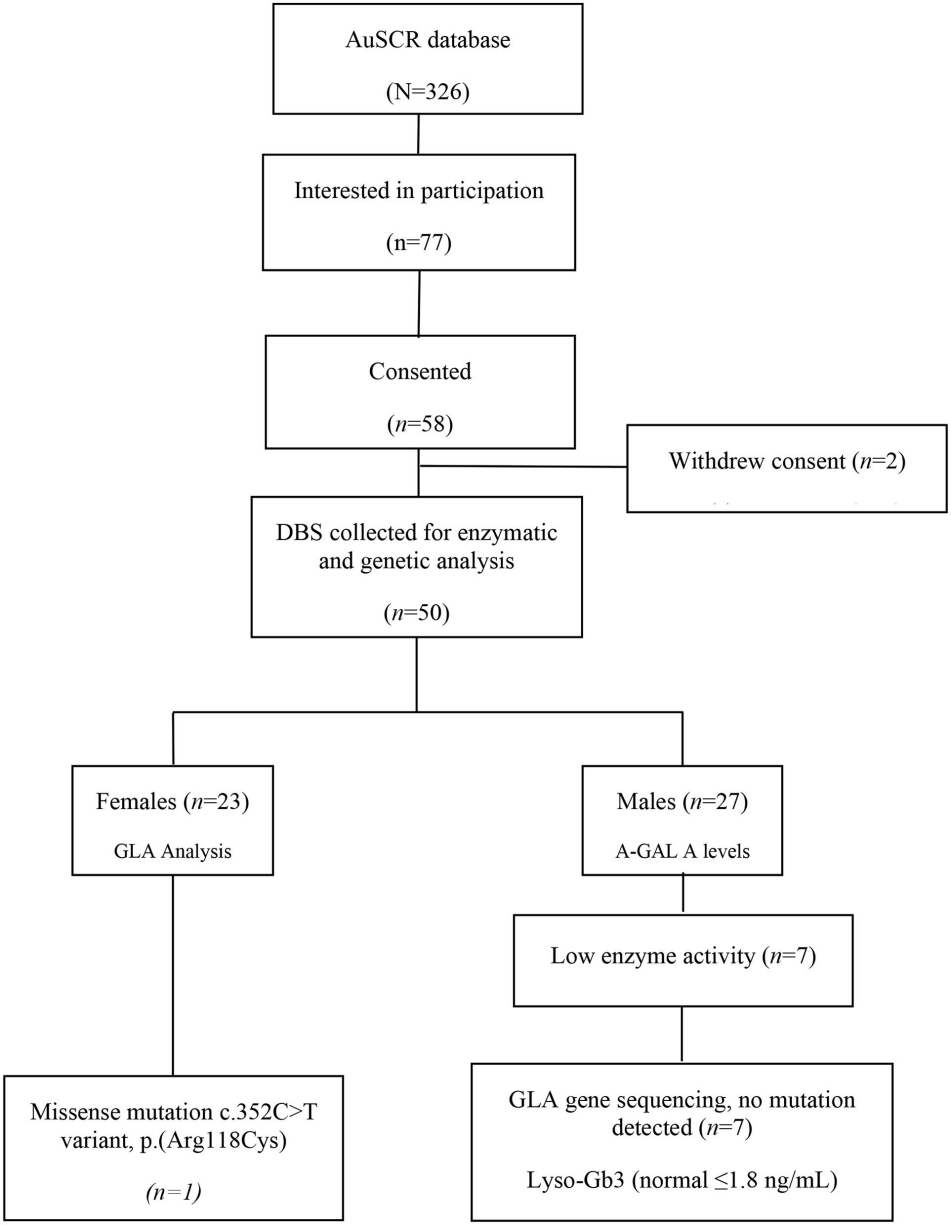
Case identification for genetic screening for Fabry disease in young stroke.

Of 326 patients who met the initial eligibility criteria in the AuSCR database and were invited to participate, 58 (18%) provided consent but six were unable to provide a blood sample and another two later withdrew their consent to provide blood samples. Thus, DBS tests for enzyme/genetic analysis were obtained from 50 participants (Figure 1). The Table 1 outlines their demographic characteristics (median age 53 years [range 19–53]; 47% female) and clinical features, with over two thirds with a history of ischemic stroke. There was a wide range of vascular risk factors and potential early manifestations of Fabry disease, and three (6%) participants reported a genetic disorder (Lynch syndrome, mitochondrial disease and Leiden thrombophilia).

**Table 1:** Demographic and clinical characteristics of young survivors of stroke.

**Variable**	**Total (*N* = 58)**
Residential area at time of stroke^a^	
Metropolitan	30 (54)
Regional	16 (29)
Rural	10 (18)
Clinical features^b^	
Age at first stroke	48 ± 8
Arrythmia	45 (88)
Proteinuria	6 (12)
Genetic disease	3 (6)
Risk factor	
Hypertension	21 (41)
Diabetes mellitus	7 (14)
Dyslipidemia	17 (33)
Smoking	8 (16)
Stroke subtype	
Ischemic	34 (67)
Hemorrhagic	7 (14)
Transient ischemic attack	9 (18)
Family history	
Genetic disease	4 (8)
Renal failure	6 (12)
Cardiac disease	35 (69)
Stroke <55 years	10 (20)
Features suggestive of Fabry disease	
Acroparesthesias^c^	37 (65)
Angiokeratomas^c^	16 (28)
Swelling and pain in joints^a^	11 (20)
Gastrointestinal symptoms^d^	41 (75)
Hearing loss^b^	45 (80)
Hypohidrosis^e^	47 (87)
Heat or cold intolerance^d^	50 (91)

*Date are n (%) or mean ± SD*.

a *Two patients with missing data*.

b *Seven patients with missing data*.

c *One patient missing data*.

d *Three patients missing data*.

e *Four patients with missing data*.

All males (27/50) had their α-GAL A enzyme level measured on DBS and, although this was low in seven, sequencing of GLA gene did not identify a clinically relevant mutation for Fabry disease. Moreover, all levels of the biomarker Lyso-Gb3 were normal (≤1.8 ng/mL) and MLPA analysis in two patients did not show any deletion or duplication.

For the 23 females, only one had a pathogenic GLA mutation c.352C>T variant, p.(Arg118Cys), despite having a normal level of Lyso-Gb3. She had a history of spontaneous intracerebral hemorrhage at the age of 49 years and reported fatigue, a history of proteinuria and cystic kidney, and a paternal history of premature vascular disease (father with chronic renal failure and brother who had a stroke at a young age) but no other risk factors. Her management included an antiplatelet agent and a statin for secondary stroke prevention.

## Discussion

In this study we identified only one potential case of Fabry disease among 326 young stroke survivors of unknown etiology who were screened in AuSCR, a Figure 1 which is consistent with other studies reporting Fabry disease in 0.5% to 3.9% of cases of cryptogenic stroke (7, 14) Similarly, large screening studies involving mixed patients with ischemic stroke, transient ischemic attack, and intracranial hemorrhage, report Fabry disease in 0.4–1% of young strokes (3, 15). Among patients who consent, the DBS test is simple, reliable and relatively rapid in obtaining a result for Fabry disease (16) in an “at-risk” patient group identified through a national prospective clinical registry.

Our study provides some support for embedding a routine genetic screening process within a national stroke registry. However, future implementation of this approach requires greater economic scrutiny over more opportunistic screening at the time of initial hospital management. AuSCR allows direct contact to be made to individuals who had expressed interest in future research, but the 18% response was lower than we had expected (17). This likely reflects our retrospective interrogation of the database for timely identification of a cohort of adequate sample size which consequently delayed the time from the index stroke event, raising the potential for compromised interest and changes to mailing addresses. Although community nurses were contracted to undertake home visits to collect the DBS samples from participants, this was for the purpose of a research project with time and funding constraints. Response rates could potentially have been higher with alternative prospective “real time” case finding, such as early coordination/communication between the AuSCR office and service providers in combination with increased awareness of Fabry disease among stroke clinicians, for detecting cases soon after the occurrence of stroke.

Although Fabry disease is relatively rare, it is important not to miss the diagnosis as enzyme replacement therapy or, more recently, oral pharmacological chaperone therapy (Migalastat) for specific mutant forms of GLA (18), are available in Australia through the Life Saving Drugs Program, a fully subsidized program for life threatening and rare diseases (19). Screening for Fabry disease also provides an opportunity for family members to be identified and managed before complications arise (7), and to be provided with appropriate expert multidisciplinary care. However, as we have not undertaken a formal economic analysis, we recognize the overall costs of screening, incorporating counseling and expensive therapy, need to be considered in the context of broader economic pressures on health care system (20).

Our study found one female with a missense GLA mutation c.352C>T, p.(Arg118Cys), which has been reported in newborns (21), patients with renal failure (22) and young stroke (14). Early studies have shown mixed results on the pathogenicity of this variant, with variable residual α-GAL A enzyme activity and Gb3 deposits in tissues (23). While further research is necessary to clarify the relevance of this variant to late-onset Fabry disease and elevated vascular risk, it is noteworthy that our female case had proteinuria, history of intracerebral hemorrhage, and family history of young stroke, complications that have been described elsewhere (5). We found an absence of genetic mutation and normal levels of Lyso-Gb3 (≤1.8 ng/mL) in seven males with low enzyme level activity. Despite the probably false-positive also reported in other studies (15), measurement of enzyme levels activity using DBS is a reliable method for screening for FD in males (7), but normal results have been reported in about one third of heterozygous females due to the random X-chromosome inactivation (24). The approach of genetic testing those with low enzyme activity is recommended to confirm the diagnosis of Fabry disease (25).

Given that as many as one-third of females with Fabry disease are missed with α-GAL A enzyme activity screening due to random X-inactivation, identification of any pathogenic GLA mutation is essential to define diagnosis and carrier status in females (10). Although Fabry disease is considered rare in heterozygous female carriers, studies have shown that they can still develop complications (8). Moreover, as we have shown with the female case in our study, this approach is more useful for screening and monitoring response to therapy as levels of Lyso-Gb3 are often within the normal range in females compared to males (26). Additionally, Lyso-Gb3 has recently been shown to be an independent risk factor for the cerebral white matter lesions in males with Fabry disease, whilst plasma lyso-Gb3 concentration correlates better with overall disease severity in females (27).

We acknowledge that our study is limited by selection bias and small sample size that has prohibited a reliable assessment of the frequency of Fabry disease in the wider population. Furthermore, we were unable to verify, or obtain further details, of the reported medical history and self-reported symptoms and signs in participants.

In conclusion, we found very few young strokes identified with probable Fabry disease through retrospective screening in a national stroke registry. Our findings draw attention to the complexities of an alternative, automated approach to screening, using a national stroke registry. Further adaption for prospective linkage to clinicians based in participating hospitals might increase case identification early after an acute event.

## Data Availability Statement

The primary data supporting the conclusions of this article will be made available by the authors, without undue reservation. The secondary use of data from the Australian Stroke Clinical Registry that partially support the findings of this study are available from the Florey Institute but restrictions apply to the availability of these data, which were used under license for the current study, and so are not publicly available.

## Ethics Statement

The studies involving human participants were reviewed and approved by Human Ethics Review Committee of Royal Prince Alfred Hospital. The patients/participants provided their written informed consent to participate in this study.

## Author Contributions

CSA, DAC, and VT: conducted the study, and responsible for the concept and design. BG and SB: contributed to the identification of participants in the AuSCR database registry according to the selection criteria. JL and AM: were responsible for the data acquisition and follow-up of test results. AM: analyzed the data and drafted the main manuscript for intellectual content. CSA, DAC, VT, JL, BG, SB, and SJ: critically reviewed the manuscript for intellectual content and elaboration of the discussion. All authors listed read and approved the final manuscript.

## Conflict of Interest

The authors declare that the research was conducted in the absence of any commercial or financial relationships that could be constructed as a potential conflict of interest. The Handling Editor declared a past co-authorship with one of the authors, VT. The reviewer MAB declared a past co-authorship with one of the authors, VT to the handling Editor.
